# Axillary Recurrence After a Tumor-Positive Sentinel Lymph Node Biopsy Without Axillary Treatment: A Review of the Literature

**DOI:** 10.1245/s10434-012-2490-4

**Published:** 2012-08-14

**Authors:** Claire M. T. P. Francissen, Pim J. M. Dings, Thijs van Dalen, Luc J. A. Strobbe, Hanneke W. M. van Laarhoven, Johannes H. W. de Wilt

**Affiliations:** 1 Department of Surgery, Radboud University Nijmegen Medical Centre, Nijmegen, The Netherlands; 2 Department of Surgery, Diakonessenhuis, Utrecht, The Netherlands; 3 Department of Surgery, Canisius Wilhelmina Hospital, Nijmegen, The Netherlands; 4 Department of Medical Oncology, Radboud University Nijmegen Medical Center, Nijmegen, The Netherlands

## Abstract

**Background:**

Sentinel lymph node biopsy (SLNB) has become standard of care as a staging procedure in patients with invasive breast cancer. A positive SLNB allows completion axillary lymph node dissection (cALND) to be performed. The axillary recurrence rate (ARR) after cALND in patients with positive SLNB is low. Recently, several studies have reported a similar low ARR when cALND is not performed. This review aims to determine the ARR when cALND is omitted in SLNB-positive patients.

**Methods:**

A literature search was performed in the PubMed database with the search terms “breast cancer,” “sentinel lymph node biopsy,” “axillary” and “recurrence.” Articles with data regarding follow-up of patients with SLNB-positive breast cancer were identified. To be eligible, patients should not have received cALND and ARR should be reported.

**Results:**

Thirty articles were analyzed. This resulted in 7,151 patients with SLNB-positive breast cancer in whom a cALND was omitted (median follow-up of 45 months, range 1–142 months). Overall, 41 patients developed an axillary recurrence. 27 studies described 3,468 patients with micrometastases in the SLNB, of whom 10 (0.3 %) developed an axillary recurrence. ARR varied between 0 and 3.7 %. Sixteen studies described 3,268 patients with macrometastases, 24 (0.7 %) axillary recurrences were seen. ARR varied between 0 and 7.1 %. Details regarding type of surgery and adjuvant treatment were lacking in the majority of studies.

**Conclusions:**

ARR appears to be low in SLNB-positive patients even when a cALND is not performed. Withholding cALND may be safe in breast cancer selected patients such as those with isolated tumor cells or micrometastatic disease.

Since the 1990s, the sentinel lymph node biopsy (SLNB) has become the standard staging procedure for patients with invasive breast cancer.^1^ According to the current treatment guidelines, treatment of the axilla in case of a negative SLNB can be safely omitted.^2^ A positive SLNB indicates that completion axillary lymph node dissection (cALND) has to be performed.^3^ In case of four or more positive lymph nodes in the dissection specimen, adjuvant radiotherapy of the supra- and infraclavicular lymph nodes is indicated.^4^ Axillary radiotherapy is possibly an appropriate alternative for cALND. In the AMAROS trial cALND and axillary radiotherapy were compared; the main objective was to show equivalent locoregional control and reduced morbidity after radiotherapy. The final results of this trial have not yet been established.^5^,^6^ Although a cALND not only provides additional prognostic information, it could also optimize regional control and potentially improve overall survival.^3^ However, in 15–20 % of the cases, a cALND leads to long-term complications such as pain, paresthesia due to intercostobrachial nerve injury, impairment of the shoulder function, or lymphedema.^7^,^8^

The axillary recurrence rate (ARR) after a cALND has been performed in patients with a positive SLNB is low. Recent publications show rates that vary between 0.2 and 0.9 % for micrometastatic disease of the sentinel lymph node (SLN) and around 1.0 % in case of macrometastatic disease.^9^–^11^ These results are comparable with results from older studies in which all patients had been treated with ALNDs.^12^ Patients with positive axillary lymph nodes are usually treated with adjuvant systemic therapy: hormone therapy, chemotherapy or both. Although this might be an important factor for the low recurrence rate in the axilla, in case of breast conservative treatment radiotherapy itself on the breast is considered an important reason for a low ARR.

Recently, several studies in patients with a positive SLNB who predominantly underwent breast conservative treatment have reported a low ARR when cALND had not been performed.^13^–^16^ A prospective randomized trial (ACOSOG Z0011) tried to investigate whether cALND could safely be omitted in these patients. The trial was closed early as a result of slow accrual. The slow accrual probably implies that in daily practice there is still concern about the safety of omitting a cALND. Because interest is growing in omitting a cALND even if the SLNB is positive, this systematic review article aims to determine the ARR after a cALND has been omitted in SLNB-positive patients.

## Methods

### Literature Search

A literature search was performed in the PubMed database using the search terms “breast cancer,” “sentinel lymph node biopsy,” “axillary” and “recurrence.” Only articles published in the past ten years and in the English language were considered. References from the selected articles were used to complete the search. Two reviewers (C.F. and P.D.) independently performed the search; potentially relevant articles were selected on the basis of the title and abstract.

### Review Inclusion and Exclusion Criteria

Articles with data regarding the follow-up of in cohorts a prospectively or retrospectively analyzed cohort of patients with SLNB-positive breast cancer, regardless of whether it was a prospective cohort or one formed retrospectively were identified. Only articles were examined that reported a cohort of patients with SLNB-positive breast carcinoma confirmed by SLNB and had follow-up data. The status of the positive SLNB was classified into three categories: micrometastatic lymph node (≤2.0 mm), macrometastatic lymph node (>2.0 mm) and not specified. Distinction between isolated tumor cells (ITC; <0.02 mm) and micrometastatic disease (0.02–2.0 mm) was not presented in all articles and these were therefore combined as micrometastatic (≤2.0 mm). Only SLNB-positive patients without a cALND were included. Cases in which it was uncertain whether cALND had been performed were excluded. Failure to report on the ARR also resulted in exclusion from this review.

Adjuvant treatment (radiation, chemotherapy or hormone therapy) was not subject to review. Therefore, failure to report on the percentage of patients receiving adjuvant treatment did not result in exclusion from this study. An effort was made, however, to extract data on adjuvant treatment when documented. Two studies reported 100 % of the observed patients had received axillary radiotherapy and were excluded. Additional information on age, clinical T stage, lymphovascular invasion and type of surgery was also collected.

Each article was reviewed independently by the two reviewers (C.F. and P.D.), and discrepancies were resolved by consensus.

### Identification of Axillary Recurrence

Axillary recurrence was defined as the detection of metastatic disease in the axilla based on a positive SLNB. Patients with concurrent breast relapse who had developed axillary disease based on a positive SLNB were not considered as having an axillary recurrence. In these instances, the axillary recurrence could reflect insufficient local control of the primary tumor. Additionally, some articles only mentioned local or locoregional recurrence. The latter were classified as having an axillary recurrence.

### Statistical Analysis

Patient and tumor related characteristics and ARRs were calculated by descriptive statistics.

## Results

A total of 216 articles were found in the PubMed database; after an intensive review of the analysis reported in the abstract, only 35 remained. The two articles reporting axillary radiotherapy in 100 % of the patients were excluded.^17^,^18^ Three research groups reported results from the same cohort in two or three different articles with a progressively longer follow-up or an increased group size. The articles with data reporting the longest follow-up were included.^19^–^21^ Therefore, 30 articles were eligible for analysis in this review.^9^,^10^,^14^–^16^,^19^–^43^

### Description of Study Data

In 30 articles results were presented for a total of 7,151 patients with SLNB-positive breast cancer in which a cALND had not been performed. Median follow-up was 45 months (range 1–142 months). One study reported an underpowered multicenter randomized clinical trial.^9^ Two studies reported results from a nationwide database.^10^,^22^ All the other series were single center reports.^9^,^10^,^14^–^16^,^19^–^43^

A cALND was not performed because of patient or clinician preference.^14^–^16^,^21^,^24^,^35^,^37^–^40^,^42^ Some studies retrospectively analyzed patients with postoperative confirmed positive SLNs, in which no secondary surgery had been performed on the axilla.^19^,^25^,^27^,^36^,^41^,^43^ In total, 41 patients developed an axillary recurrence without evidence of ipsilateral breast cancer recurrence. The ARR varied between 0 and 7.1 % in the different studies.

### ARR After Micrometastases/ITCs are Found in the Sentinel Node

We further classified patients on the basis of the sentinel node status. In a total of 27 studies 3,468 patients were identified with micrometastatic disease in the sentinel node who had not undergone a cALND (Table 1). Median follow-up was 42 months; 10 patients (0.3 %) developed an axillary recurrence. The axillary recurrences were reported to occur in 3 patients who underwent breast-conserving therapy (BCT) and in 2 patients who underwent mastectomy. In 5 patients an axillary recurrence was reported, but details regarding the type of breast surgery was not mentioned in the article. Of the 10 patients, two were treated with adjuvant radiotherapy and one without. In the other patients, only percentages of radiotherapy in the total group of patients were given (21 % up to 72 %) or radiotherapy was not mentioned at all.^9^,^10^,^22^–^24^,^33^,^35^

**Table 1:** Sentinel node status: ‘micrometastatic’ disease (micrometastases (0.02–2.0 mm) and ITC (<0.02 mm))

Study	Year	No. patients	Median age (y)	T1 (%)	LVI present (%)	Breast-conserving therapy (%)	Chemo/hormone therapy (%)	Adjuvant RT axilla (%)	Follow-up (mo), median (range)	Axillary recurrence, *n* (%)
Yi et al.^22^	2010	1,767	61	69	NM	79^b^	NR	NM	50^a^	2 (0.1 %)^a,b^
Giuliano et al.^9^	2010	160	54	70	36	100	60/48^b^	NM	76	1 (0.9 %)^a^
Takei et al.^19^	2010	100	55	30	78	92^b^	19/77^b^	52^b^	58^a^	0
Cyr et al.^23^	2010	61	57	72	21	84	67^a^	NM	60 (2–112)	1 (1.6 %)
Yegiyants et al.^24^	2010	33	57	66	43	100^b^	92/76^b^	0	79 (6–142)	1 (3.0 %)
Meretoja et al.^25^	2010	48	67	NM	NM	44^b^	16/75^b^	13^b^	37 (9–78)	0
Degnim et al.^26^	2010	50	57	70	0	100	64	14	38 (<1–104)^a^	0
Pugliese et al.^27^	2010	76	59	NM	21	72	63/90	4.6	76.8	0
Bilimoria et al.^10^	2009	530	58^a^	63^a^	NM	81^b^	71/74^b^	NM	64 (60–72)	3 (0.6 %)
Pernas et al.^28^	2009	45	55	NM	9	100	100	NM	60 (8–94)	0
Langer et al.^20^	2009	27	62	72^a^	NM	52	20/76^c^	NM	77 (24–106)	0
Zakaria et al.^29^	2008	69	62	62	29	60^b^	53/87^b^	19^b^	30 (3–66)	0
Cox et al.^30^	2008	25	59	72	68	NM	36/32	NM	20.4 (12–84)	0
Hwang et al.^16^	2007	157	56	72	22	69^b^	56/27^b^	58^b^	30 (1–62)	0
Schulze et al.^31^	2006	5	64^c^	100^a^	0	74^b^	3/68^a^	NM	49 (+/NM 17)^a^	0
Haid et al.^32^	2006	8	59^a^	77^a^	NM	87^b^	32/93^a^	NM	47 (7–90)	0
Swenson et al.^33^	2005	63	59^a^	82^a^	NM	75^a^	42/58^a^	NM	33 (2–73)	1 (1.6 %)
Schrenk et al.^34^	2005	16	59^a^	61^a^	NM	78^a^	NR	0	48	0
Jeruss et al.^15^	2005	73	58^a^	57^a^	NM	74^a^	85/70^a^	NM	28 (1–98)	0
Fan et al.^35^	2005	27	53^a^	71	28	NM	NR	63	31 (6–76)	1 (3.7 %)
Chagpar et al.^36^	2005	15	57^a^	89^a^	2	86^a^	33	NM	40 (1–54)	0
Carlo et al.^37^	2005	20	57^a^	84^a^	NM	92^a^	100	NM	60^a^	0
Fournier et al.^38^	2004	6	53^a^	74	NM	NM	NR	NM	12 (3–30)	0
Guenther et al.^14^	2003	39	62	67	NM	NM	100	2	32 (4–61)	0
Ganaraj et al.^39^	2003	17	53^a^	59	46	NM	NR	NM	30	0
Fan et al.^40^	2003	27	NM	81	NM	100	100	3	30 (21–48)	0
Liang et al.^41^	2001	4	55^a^	NM	NM	NM	53/47	NM	14 (1–27)	0

*SLNB* sentinel lymph node biopsy, *LVI* lymphovascular invasion, *RT* radiotherapy, *NR* not reported (patients received systemic therapy, but the exact percentage was not reported), *NM* not mentioned in the article, *SN* sentinel node

*SN status* micrometastatic disease, micrometastases (0.02–2.0 mm; and ITC (<0.02 mm)

^a^Total group (not specified separately for SN-positive patients)

^b^Not specified separately for SN status

^c^For all SN, including both SN negative and positive

Two of the 27 studies investigated national databases. From the Surveillance, Epidemiology, and End Results database, Yi et al.^22^ identified 26,986 patients with disease-positive lymph nodes, of which 4,425 (16.4 %) had only undergone SLNB. After nodal evaluation with lymph node count threshold, i.e. patients were considered only to have undergone a SLNB if they had had five or fewer nodes examined, 3,240 patients remained with a median age of 60 years (range 24–96 years). Of these patients, 1767 had micrometastatic disease in the SLN and an ipsilateral regional event was seen in 0.1 %.

Bilimoria et al.^10^ identified 97,314 patients in the United States National Cancer Data Base with clinically node-negative breast cancer who had had a positive sentinel node. Of these, 20.8 % had undergone a SLNB without a cALND. Only patients diagnosed between 1998–2000 with a follow-up reported between 2004 and 2006 were used in the outcome analysis; 1,988 (9.8 %) had only undergone SLNB. The median age was 58 years (range 49–69 years), median follow-up was 64 months. Axillary recurrences were seen in 0.6 % of the 530 patients with microscopic nodal metastases.

The 25 other studies reported on smaller cohorts in which the ARR varied between 0 and 3.7 %. The median age was 58 years (range 53–67 years), most patients had undergone BCT (44–100 %) and had received some form of adjuvant systemic therapy (36–100 %). Nine studies reported on radiotherapy of the axilla in 2–63 % of patients.^14^,^16^,^19^,^25^–^27^,^29^,^35^,^39^

### ARR After Macrometastases are Found in the Sentinel Node

Table 2 shows 16 studies that described patients with macrometastatic disease in the SLN in whom a cALND had not been performed. A total of 3,268 patients were identified, with a median follow-up of 43 months (range 1–142 months). Twenty-four axillary recurrences were observed (0.7 %). In this group the median age was 58 years (range 53–64 years). The national database study by Yi et al.^22^ showed an ARR of 0.1 % in 1,473 patients with macrometastatic disease; Bilimoria et al.^10^ found an ARR of 1.2 %. The studies that reported on smaller cohorts showed an ARR between 0 and 7.1 %. The latter was observed in a study with only 14 patients. In case of macrometastatic disease in the sentinel node, 6 studies reported that patients had been treated with axillary radiotherapy in 2–63 % of the patients.^14^,^16^,^19^,^29^,^35^,^40^ In the majority of patients who developed an axillary recurrence (*n* = 24) details regarding the type of surgery of the primary tumor were lacking. Three of the patients in this group were treated with BCT. Detailed information regarding adjuvant radiotherapy was also lacking. Of the 24 patients with an axillary recurrence, 3 patients received radiotherapy. In the other patients, only percentages of radiotherapy in the total group of patients were given (21 % up to 72 %).^9^,^10^,^22^,^24^

**Table 2:** Sentinel node status: macrometastatic disease (>2.0 mm)

Source	Year	No. patients	Median age (y)	T1 (%)	LVI present (%)	Breast-conserving therapy (%)	Chemo/hormone therapy (%)	Adjuvant RT axilla (%)	Follow-up (mo), median (range)	Axillary recurrence, *n* (%)
Yi et al.^22^	2010	1,473	61	69	NM	79^b^	NR	NM	50^a^	3 (0.2 %)
Giuliano et al.^9^	2010	199	54	70	36	100	60/48^b^	NM	76	2 (0.9 %)^b^
Takei et al.^19^	2010	32	55	30	78	92^b^	19/77^b^	52^b^	58^a^	0
Yegiyants et al.^24^	2010	14	57	66	43	100	92/76^b^	0	79 (6–142)	1 (7.1 %)
Bilimoria et al.^10^	2009	1,458	58^a^	63^a^	NM	81^b^	71/74^b^	NM	64 (60–72)	18 (1.2 %)
Zakaria et al.^29^	2008	17	62	62	29	60^b^	53/87^b^	19^b^	30 (3–66)	0
Hwang et al.^16^	2007	39	56	72	22	69^b^	56/27^b^	58^b^	30 (1–62)	0
Schulze et al.^31^	2006	1	64^c^	100^a^	0	74^b^	3/68^a^	NM	49 (+/NM 17)^a^	0
Haid et al.^32^	2006	2	59^a^	77^a^	NM	87^b^	32/93^a^	NM	47 (7–90)	0
Swenson et al.^33^	2005	4	59^a^	82^a^	NM	75^b^	42/58^a^	NM	33 (2–73)	0
Schrenk et al.^34^	2005	4	59^a^	61^a^	NM	29^b^	NR	0	48	0
Fan et al.^35^	2005	11	53^a^	71	28	NM	NR	63^b^	31 (6–76)	0
Chagpar et al.^36^	2005	1	57^a^	89^a^	2	86^b^	33^b^	NM	40 (1–54)	0
Carlo et al.^37^	2005	2	57^a^	84^a^	NM	92^a^	100	NM	60^a^	0
Guenther et al.^14^	2003	7	62	67	NM	NM	100	2^b^	32 (4–61)	0
Fant et al.^40^	2003	4	NM	81	NM	NM	100	3^b^	30 (21–48)	0

*SLNB* sentinel lymph node biopsy, *LVI* lymphovascular invasion, *RT* radiotherapy, *NR* not reported (patients received systemic therapy, but the exact percentage was not reported), *NM* not mentioned in the article, *SN* sentinel node

*SN status* macrometastatic disease (>2.0 mm)

^a^Total group (not specified separately for SN-positive patients)

^b^Not specified separately for SN status

^c^For all SN, including both SN negative and positive

Table 3 shows four studies that reported SLNB positivity, but which did not describe the exact sentinel node status. A total of 409 patients with a median follow-up of 55 months were identified. The ARR varied between 0 and 2.1 %, and a total of 7 axillary recurrences had been noted. One study included patients receiving axillary radiotherapy; however, this applied to only 15 % of the patients.^21^

**Table 3:** Sentinel node status: not specified

Source	Year	No. patients	Median age (y)	T1 (%)	LVI present (%)	Breast-conserving therapy (%)	Chemo/hormone therapy (%)	Adjuvant RT axilla (%)	Follow-up (mo), median (range)	Axillary recurrence, *n* (%)
Giuliano et al.^9^	2010	66	54^b^	70	36	100	60/48^b^	NM	76	1 (0.9 %)^b^
Geertsema et al.^43^	2010	39	60^a^	64^a^	NM	NM	NM	NM	40^b^	0
Park et al.^21^	2007	287	59	78	22	68	NR	15	23 (6–87)	6 (2.1 %)
Calhoun et al.^42^	2005	17	64	65	NM	NM	88	NM	81^a^	0

*SLNB* sentinel lymph node biopsy, *LVI* lymphovascular invasion, *RT* radiotherapy, *NR* not reported (patients received systemic therapy, but the exact percentage was not reported), *NM* not mentioned in the article, *SN* sentinel node

*SN status* not specified

^a^Total group (not specified separately for SN-positive patients)

^b^Not specified separately for SN status

## Discussion

Recent studies have questioned the need for a cALND in patients with a positive SLNB because the ARR is relatively low if cALND has not been performed. In daily practice, a shift toward omitting the cALND in these patients is taking place, especially in those patients with micrometastatic disease. Results from two large database studies in the US demonstrate that already 16.4–20.8 % of the patients with a positive sentinel node did not undergo axillary treatment.^10^,^22^ The present review demonstrates low ARRs in studies which included at least 100 patients for patients who did not undergo a cALND although they had a tumor-positive SLNB.^9^,^10^,^16^,^18^,^22^ The rates varied between 0 and 0.9 % for micrometastatic disease and 0.2 to 1.2 % for macrometastatic disease. These rates are comparable to those seen in patients with a positive SLNB in which a cALND had been performed (recurrence rates between 0.2 and 1.0 %).^9^,^10^ The rates found are also of the same magnitude as those for patients with a negative SLNB (without cALND) for whom the ARR ranged from 0.3 to 1.4 %.^11^,^44^

In this review we included the preliminary results from the ACOSOG Z0011 trial. This is the only prospective trial in which patients with sentinel node metastases were randomized to undergo axillary lymph node dissection after SLNB versus SLNB without specific axillary treatment.^9^ All these patients were treated with BCT and tangential field whole breast irradiation. In addition, the majority of the patients (97 %) in that study received adjuvant systemic treatment. At a median follow-up of 6.3 years (interquartile range 5.2–7.7), no significant difference was seen in the ARR between the two groups (0.5 % in the cALND group and 0.9 % in the group without cALND). Moreover, overall survival was not different in both study arms.^45^ Unfortunately, the trial was underpowered and closed early because accrual was low and the ARR was lower than expected in both arms.^9^,^45^

In addition to the low chance of recurrence shown by the ARR, there are other arguments to eliminate a cALND for selected patients. First of all, in the current guidelines adjuvant systemic therapy is advised on the basis of tumor and patient characteristics and axillary lymph node involvement. Treatment recommendations are rarely altered by the additional information gained by the cALND results. Geertsema et al.^43^ reported additional tumor positive axillary lymph nodes in 16 % of the patients undergoing a cALND after a negative frozen section. None of those patients would have received additional systemic therapy on the basis of the cALND findings. The AMAROS trial compared cALND with axillary radiotherapy in positive sentinel node patients. It also demonstrated that information obtained from cALND rarely altered treatment recommendations made before the cALND.^4^,^6^ Although patients with four or more positive lymph nodes are supposed to receive adjuvant radiotherapy on the axilla and supraclavicular region, that was only necessary in 12 % of the patients in the trial.^6^,^46^ Most probably the majority of patients with four or more positive lymph nodes were preoperatively identified by palpation or ultrasound as being node positive and did not undergo SLNB as such patients routinely undergo an axillary dissection at the same time as treatment for the primary tumor.

Secondly, only in 10–20 % of patients with ITCs and micrometastases and around 40 % of patients with macrometastases does the cALND contains additional lymph node metastases.^6^,^47^ Several nomograms have been developed to predict the chance of additional lymph node metastases; however, each has limitations.^48^,^49^ Because in the general population the majority of additional lymph nodes are negative, performing a cALND would have no therapeutic value or additional staging effects.

Thirdly, the effect of additional systemic therapy in patients with node positive disease is an important issue. A few decades ago Canabes et al.^50^ had reported that the ARR in patients with tumors smaller than 3 cm was only 2 % if the axilla had not been treated. That study compared axillary dissection to no axillary dissection. However, that occurred in an era in which only a minority of the patients were treated with adjuvant systemic therapy. Now that modern systemic therapy is given in almost all patients with a positive SLNB, the recurrence rate now that can be expected will most probably be lower. The MIRROR trial studied the effects of systemic therapy in patients with ITCs or micrometastases regardless of axillary treatment; it demonstrated an improvement of disease-free survival in patients who had received adjuvant systemic therapy.^51^ In neo-adjuvant chemotherapy protocols, not only is the size of the tumor substantially reduced but also the size of lymph node metastases.^52^ Pathological complete response by the primary tumor has been demonstrated to be highly predictive of pathological complete response in the axillary nodes.^53^ Charfare et al.^54^, Vlastos et al.^55^ and Kuerer et al.^56^ reported a pathologically complete response in 3–34 % of the patients. Thus, patients with a positive lymph node status at initial evaluation, can be considered to have a negative axillary nodal status as a result of neo-adjuvant chemotherapy.^54^–^56^ This effect might also be of importance in adjuvant chemotherapy protocols. Therefore, potentially positive lymph nodes in the axilla could be sterilized by chemotherapy, which would then reduce the theoretical ARR.

Fourthly, not only systemic therapy but also adjuvant radiotherapy can be regarded as a treatment modality for any remaining axillary lymph node metastases. Radiotherapy to the breast as part of BCT includes the lowest portion of the axilla. In several studies it has been objectified that the clip marking the SLN fell within the standard tangential fields of the whole breast radiotherapy in 78–94 % of the patients.^57^,^58^ Veronesi et al.^59^ described that radiotherapy to the breast is one of the possible explanations for the lower than expected numbers of axillary metastases in the no axillary radiotherapy arm of their randomized trial to assess the role of axillary radiotherapy. A recently published systematic review demonstrated that patients with a node negative SLN who underwent radiotherapy had a significantly lower rate of axillary recurrences as compared to patients who had not been not treated with postoperative radiotherapy of the breast.^60^

A limitation of this review is the fact that all but one of the reviewed studies were retrospectively analyzed. It is therefore difficult to control for treatment bias and selection bias. In some studies patients were more likely to be older and have more favorable tumors characteristics such as smaller size, low tumor grade and less lymphovascular infiltration than in the general breast cancer population. This is shown in Table 4 by the trend for smaller tumor size, more BCT and micrometastases in patients who has been treated with only SLNB. Unfortunately, details on the type of surgery or adjuvant radiotherapy for those patients who developed an axillary recurrence was not presented in the majority of the different studies. Axillary recurrences were reported in 6 patients after BCT and 2 after mastectomy, whereas in 28 patients it was not described in detail. Also, the results extracted from the two American database studies dominated the results of this review because of the greater number of patients in comparison with the other 28 studies. In those database studies the selection bias is substantial because patients were selected to undergo only SLNB if they were more likely to have a favorable prognosis. Also, these cancer registries may underreport axillary recurrences because the definition “ipsilateral regional events” is used instead of “axillary recurrence.” Although the other studies do use the exact definition, underreporting might still occur in all studies because of their retrospective nature.

**Table 4:** Patients, tumor and treatment characteristics in studies with >100 patients who underwent SLNB only for positive breast cancer, compared with patients who underwent ALND

Favorable characteristic	Study^a^	ALND	SLNB only
Tumor size <2 cm (%)	Yi et al.	50.4	68.6
	Giuliano et al.	67.9	70.6
	Takei et al.	x	29.5
	Bilimoria et al.	49.1	62.9
	Hwang et al.	x	72.4
	Park et al.	62	78
Radiotherapy (%)	Yi et al.	53.6	59.9
	Giuliano et al.	NM	NM
	Takei et al.	x	84.8
	Bilimoria et al.	72.1^b^	72.1^b^
	Hwang et al.	x	58.2
	Park et al.	NM	15
Median age (y)	Yi et al.	55	60
	Giuliano et al.	56	54
	Takei et al.	x	55
	Bilimoria et al.	56	58
	Hwang et al.	x	56
	Park et al.	52	59
Breast-conserving surgery (%)	Yi et al.	53.9	78.7
	Giuliano et al.	100	100
	Takei et al.	x	92.4
	Bilimoria et al.	49.6	81.4
	Hwang et al.	x	68.9
	Park et al.	55	68
Micrometastases (%)	Yi et al.	17.2	54.5
	Giuliano et al.	37.5	44.8
	Takei et al.	x	56.8
	Bilimoria et al.	8.5	18.2
	Hwang et al.	x	45.9
	Park et al.	NM	NM
No. of LNs removed, median (range)	Yi et al.	15 (9–64)	2 (1–5)
	Giuliano et al.	17	2
	Takei et al.	x	3 (1–11)
	Bilimoria et al.	15 (12–20)	2 (1–3)
	Hwang et al.	x	3 (1–14)
	Park et al.	>10	NM (1–9)

*SLNB* sentinel lymph node biopsy, *ALND* axillary lymph node dissection, *LN* lymph node, *NM* not mentioned in the article, *SN* sentinel node, *x* = no comparison with a group patients treated with ALND is made

^a^Studies are as follows: Yi et al.^22^, Giuliano et al.^9^, Takei et al.^19^ (total group, not specified separately for SN-positive patients), Bilimoria et al.^10^, Hwang et al.^16^ (total group, not specified separately for SN-positive patients), and Park et al.^21^ (in ALND 95.7 % > 10 nodes excised, in SLNB 85 % 1–9 nodes excised)

^b^After breast-conserving surgery

The mean follow-up in the studies in this review ranged between 1 and 142 months, which is relatively short for drawing definite conclusions about axillary recurrence. However, studies with long-term follow-up have shown that the majority of locoregional recurrences, including axillary relapses, occur within five years of the initial treatment and most axillary recurrences (approximately 90 %) are evident within the first 2 or 3 years after surgery. This compares well to the median follow-up for the present study.^61^,^62^

Heterogeneity among the included studies was substantial and is a major drawback of this review. Although BCT was performed in the majority of patients for all reviewed studies, the results in each individual study were not given separately for BCT versus mastectomy. With regard to adjuvant treatment, most of the studies reported that adjuvant treatment was given according to the national guidelines, but the adjuvant treatment was not specified for those patients with a recurrence. Radiotherapy on the axilla was also given in 10 of these studies but a substantial number of patients in each study did not receive that therapy. This is an important issue because the MA.20 preliminary report demonstrated a reduction in locoregional recurrence when the axilla has been irradiated.^63^ This makes it difficult to form definite conclusions about the influence of adjuvant systemic therapy and radiotherapy on the recurrence rate. The absence of the distinction between micrometastases and ITCs in some of the studies is also a drawback because patients with ITCs in the SLNB are nowadays classified as “node-negative patients,” not requiring a cALND. Recently Jakub et al.^64^ suggested that not the number of positive SLN, but the number of positive non-SLNs is important for prognosis. Details about this issue of additional non-SLN positivity are lacking in the reviewed studies.

Lastly, the definition and surgical technique of the SLNB itself is a limitation. In daily routine most patients have one or two SLNs, but in the six studies with 100 or more patients in this review mean number of biopsied nodes is 2–11 nodes. In the two large American databases, before analysis with lymph node count threshold (five or fewer nodes examined) 4–16 nodes and 1–44 nodes were included as SLNB patients, which should probably be considered as complete axillary lymph node dissections.

Overall survival was not subject of this review, although it is clinically relevant to investigate whether omission of cALND affects survival. Yi et al.^22^ stated that compared with SLNB alone, cALND does not seem to be associated with improved survival in patients with micrometastasis in the SLNB. Bilimoria et al.^10^ described that, in case of micrometastases, a cALND does not appear to improve the overall outcome, as there were no significant differences in survival or in axillary recurrences. In case of macrometastatic disease, there was a nonsignificant trend toward better outcomes in axillary recurrences and survival after cALND, but only after analysis was adjusted for clinicopathologic differences.^10^ In the ACOSOG Z0011 trial, Giuliano et al.^65^ observed no differences in overall survival between the cALND arm and the arm in which cALND was not performed. Long-term results and the impact of the omission of axillary lymph node dissection on survival are awaited.

In conclusion, this review shows that the ARR is low in patients with a positive SLNB, even if a cALND is not performed. Recent efforts to develop nomograms in order to predict which patients should undergo a cALND, fails to identify patients who really benefit of this procedure. On the contrary, avoiding a cALND seems to be safe in the majority of SLNB-positive patients, especially in case of ITCs or micrometastatic disease. In patients who meet the Z0011 trial criteria and undergo adjuvant radiotherapy and chemotherapy, cALND has little to no effect on local recurrence and survival. However, large (prospective randomized) studies are lacking for patients with a potentially higher risk for regional recurrences such as multiple macrometastases in the SLN, patients with larger primaries, patients who underwent neoadjuvant chemotherapy or patients undergoing mastectomy or BCT without radiotherapy. Such randomized trials or large clinical cohort studies are necessary to investigate the long-term effects on axillary recurrences and survival of omitting cALND in high risk patients.^66^,^67^ In the future, cALND will probably be used as an effective treatment procedure rather than a procedure to prevent axillary recurrence.^68^
